# Recent Advances in Scaling up Bioelectrochemical Systems: A Review

**DOI:** 10.3390/biotech14010008

**Published:** 2025-01-31

**Authors:** Diego A. Corona-Martínez, Silvia Y. Martínez-Amador, José A. Rodríguez-De la Garza, Elan I. Laredo-Alcalá, Pedro Pérez-Rodríguez

**Affiliations:** 1 Departamento de Ciencias del Suelo, Universidad Autónoma Agraria Antonio Narro, Calzada Antonio Narro 1923, Buenavista, Saltillo 25315, Coahuila, Mexico; diegoa.coronam@uaaan.edu.mx; 2 Departamento de Botánica, Universidad Autónoma Agraria Antonio Narro, Calzada Antonio Narro 1923, Buenavista, Saltillo 25315, Coahuila, Mexico; silvia.martinez@uaaan.edu.mx; 3 Departamento de Biotecnología, Facultad de Ciencias Químicas, Universidad Autónoma de Coahuila, José Cárdenas Valdez y Venustiano Carranza S/N, Colonia República Oriente, Saltillo 25280, Coahuila, Mexico; antonio.rodriguez@uadec.edu.mx; 4 Centro de Investigación para la Conservación de la Biodiversidad y Ecología de Coahuila, Universidad Autónoma de Coahuila, Miguel Hidalgo 212, Zona Centro, Cuatrociénegas 27640, Coahuila, Mexico; elan_laredo@uadec.edu.mx

**Keywords:** microbial fuel cells, microbial electrolysis cells, electrode materials, energy recovery, power density

## Abstract

Bioelectrochemical systems (BESs) are devices capable of converting chemical energy into electrical energy using microorganisms as catalysts. These systems have been extensively studied at the laboratory level, but, due to multiple difficulties, their large-scale implementation has been explored only sparingly. This study presents the most recent technological advances for scaling up BESs. In the same way, the main technical and economic challenges that hinder the correct implementation of these systems at a large scale are mentioned. The study concludes with a review of successful case studies in scaling up BESs and discusses future directions and emerging trends.

## 1. Introduction

Bioelectrochemical systems (BESs) are a highly complex emerging technology with the capacity to produce bioenergy and recover high-value-added products (e.g., nutrients, H_2_, and CH_4_) [1]. These systems transform the chemical energy stored in different substrates (glucose, acetate, etc.) into electrical energy through microorganisms acting as catalysts. These microorganisms, commonly known as exoelectrogens, can transfer electrons from their cells to an external electron acceptor, such as an electrode in a bioelectrochemical system [2]. Since their discovery in 1911 [3], BESs have been applied in contaminated soil remediation [4,5,6], greenhouse gas mitigation [7,8,9], biosynthesis [10,11,12], and wastewater treatment [13,14,15].

Scaling up these systems is imperative for their effective implementation in real-world settings. This will enhance the treatment capacity of contaminated effluents (municipal and industrial), boost energy production, and facilitate resource recovery [16,17,18]. However, the scale-up of BESs poses significant challenges, including high production and operating costs, low energy efficiency, and poor microbial community stability [19,20].

Recently, multiple strategies have emerged to address these challenges, incorporating advances in materials science, microbial genetic engineering, and process optimization to promote the scalability of BESs. These innovations include developing new electrode materials, producing novel biocatalysts (electroactive microorganisms), and optimizing the operating conditions to promote microbial activity [21,22].

This paper thus summarizes the most recent advances in scaling up bioelectrochemical systems. It also defines the main technical and economic challenges associated with this process. Finally, the paper presents the future directions and emerging trends for the large-scale implementation of such systems.

## 2. Fundamentals of Bioelectrochemical Systems

### 2.1. Basic Principles of BESs

A BES harnesses the capacity of specific microorganisms, designated as exoelectrogens, to degrade organic compounds, a process that concomitantly releases electrons. In contrast to the conventional behavior of electrons within biological reactions, which typically remain inside the cell, these exoelectrogens can transfer them to an external medium, such as the electrodes of a BES [23,24]. This process, known as extracellular electron transfer (EET), is a critical element of these systems. To date, several mechanisms by which exoelectrogens can transfer electrons to electrodes have been identified [25,26]:

- Short-range direct electron transfer (SR-DET): through redox proteins such as outer membrane cytochromes;
- Indirect electron transfer (IET): through redox mediators secreted by exoelectrogens, such as flavins produced by the genus *Shewanella*;
- Long-range direct electron transfer (LR-DET): through electrically conductive appendages, such as nanowires.

**Figure 1:**
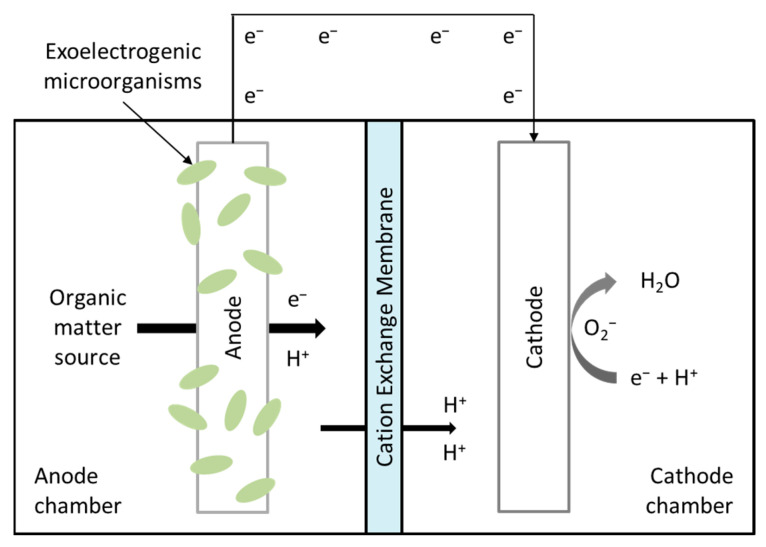
Schematic representation of a basic BES.

A BES comprises two chambers: an anode chamber and a cathode chamber. Within the anode chamber, exoelectrogens oxidize organic matter, thereby producing electrons and protons. The electrons flow from the anode to the cathode through an external circuit, generating electrical energy. The protons pass through a cation exchange membrane into the cathodic chamber to maintain ionic equilibrium. In the cathodic chamber, electrons and protons react with an electron acceptor, such as oxygen, to complete the circuit, forming water as the final product [27,28,29,30]. This process is illustrated in Figure 1.

### 2.2. Types of BES

**Figure 2:**
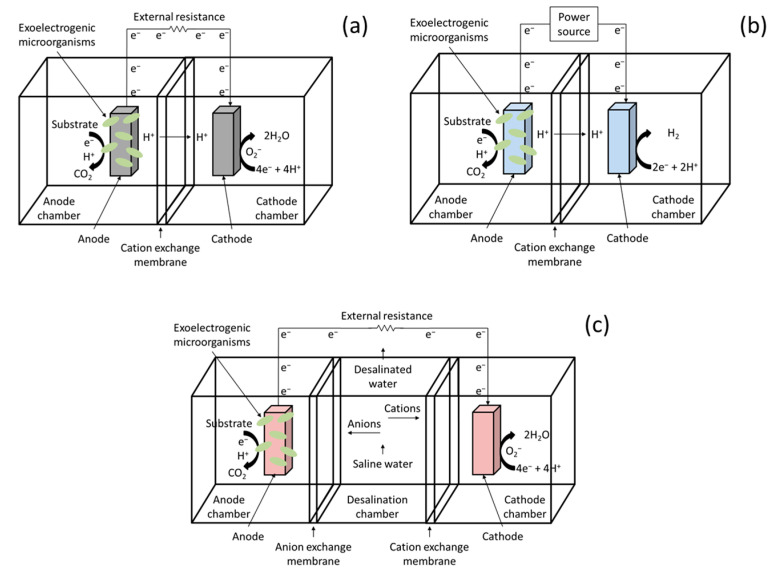
Schematics of different types of bioelectrochemical systems: (**a**) MFC; (**b**) MEC; (**c**) MDC.

A broad array of BESs exists, each focused on a particular purpose. The most notable include the following:

- Microbial fuel cells (MFCs, Figure 2a) are a type of BES that focuses on generating electricity from the oxidation of organic matter by microbial action. These systems can be configured in a variety of structural designs, including single-chamber configurations, where the anode and cathode are positioned within a single chamber separated by a cation exchange membrane, and dual-chamber configurations (most commonly used in laboratory scale experiments), where the anode and cathode are situated in separate chambers, with an ion exchange membrane acting as an isolator between them. MFCs find application in the remediation of contaminated water and soil while generating electrical power, as biosensors to detect the presence of toxic compounds or to assess the quality of wastewater, and to generate hydrogen when coupled to microbial electrolysis cells [31,32];
- Microbial electrolysis cells (MECs, Figure 2b) are devices that facilitate the conversion of organic substrates to hydrogen and other products of commercial interest (methane, acetate, etc.) through microbial metabolism and the application of an external voltage. MECs resemble MFCs in that both rely on electrically active bacteria on the anode surface to convert organic matter into protons, electrons, and carbon dioxide. However, MECs require a power source to overcome the thermodynamic hurdle to enable the synthesis of the final product. Therefore, MECs are used not only in microbial electrosynthesis but also in wastewater treatment (by oxidizing organic matter in the anode chamber) and metal recovery (by facilitating the reduction and precipitation of these ions) [33,34];
- Microbial desalination cells (MDCs, Figure 2c) are a bioelectrochemical system (BES) that utilizes the chemical energy stored in wastewater and other organic-rich waste to desalinate salt water. This process uses an electrical current generated by exoelectrogenic bacteria, which facilitates the migration of ions through anion and cation exchange membranes. The design of this type of system consists of three chambers: an anode chamber, a cathode chamber, and a desalination chamber, separated by ion exchange membranes. The primary benefits of this type of system include its low energy consumption (due to the implementation of microbiological activity as a catalyst), its high sustainability, and its capacity for the simultaneous treatment of pollutants [35,36].

### 2.3. Key Components of BESs

In the pursuit of the optimal design and construction of BESs, the following components require particular consideration:

- Anode: This component functions as the electrode where exoelectrogens execute the oxidation of the organic matter present in the substrate. Furthermore, it functions as the support on which the electroactive microorganisms adhere, thereby facilitating biofilm development. The anode is essential because of its capacity to act as an electron acceptor for microorganisms under conditions of anaerobiosis in the system. Several factors, including the conductivity, surface area, and biocompatibility of the manufacturing material, influence the anode’s efficiency. Consequently, carbonaceous materials (e.g., carbon felt, carbon mesh, carbon cloth, and graphite brush) are optimal for such applications [37,38,39];
- Cathode: The electrode that receives the electrons generated by the oxidation of organic matter at the anode. These electrons are then utilized to reduce oxygen and other electron-accepting compounds, such as nitrites, nitrates, and sulfates. Depending on the configuration of the BESs, the cathode can promote the formation of products such as hydrogen, biopolymers, and other chemicals. The cathode can be classified as either biotic or abiotic, depending on the operational design of the BES. Biotic cathodes utilize electroactive aerobic microorganisms to catalyze reduction reactions, whereas abiotic cathodes employ precious metals, such as platinum, to catalyze analogous reactions. In systems such as MFCs, the efficiency of the cathode can significantly impact the amount of electrical power generated. [40,41];
- Cation exchange membrane (CEM): This constitutes a type of semipermeable membrane characterized by negatively charged functional groups (SO_3_^2−^, COO^−^, PO_3_^2−^, HPO_3_^−^, etc.) that facilitate the selective transport of cations across the membrane while impeding the movement of anions. In BESs, they separate the anodic and cathodic chambers, allowing the transport of protons (and other cations present in the substrate) generated at the anode surface to the cathode. This process is indispensable for maintaining the electroneutrality of the system. Also, CEMs minimize the diffusion of oxygen from the cathodic chamber to the anode chamber, which, if not avoided, would considerably decrease the system’s performance. To date, Nafion^TM^ 117 (DuPont), CMI-7000 (Membranes International Inc., Ringwood, NJ, USA), and Flemion™ (Asahi Glass, Tokyo, Japan) membranes are the most widely used in BESs due to their high ionic conductivity and permselectivity [42,43,44];
- Electroactive microorganisms (EAMs): These function as catalysts, facilitating redox reactions using electrodes as electron acceptors (anode) or donors (cathode). The role of EAMs in BESs is contingent on the type of system and the microorganism selected. Exoelectrogens have been identified in all three domains of biological classification, including bacteria, archaea, and eukaryotes. Examples of well-studied exoelectrogens include *Geobacter sulfurreducens*, *Shewanella oneidensis*, *Pseudomonas aeruginosa,* and *Escherichia coli* [45,46];
- Substrate: This is defined as the source that provides the organic matter that EAMs oxidize to generate electrons. The nature (chemical composition and biodegradability) and concentration (low and high substrate concentrations can inhibit the growth of exoelectrogens) of the substrate largely define the efficiency and performance of the BES. The substrate can range in complexity, depending on the demands of the specific process and the microbial density and diversity. Examples of substrates utilized as fuel in BESs include glucose, acetate, lactate, wastewater (domestic and industrial), solid waste (sludge, food waste, and lignocellulosic biomass), and gaseous substrates (CO and CO_2_), among others [47,48,49,50].

### 2.4. Performance of BESs

BES performance can be assessed using various measurements and parameters:

- Current density is a measure of the amount of electrical current flowing through a given area. Current density determines the rate at which energy is recovered in the system. This measure is calculated by dividing the electric current by the projected area of the anode and is expressed in amperes per square meter (A m^−2^) [51];
- Power density is a measure of the amount of energy that can be extracted from a BES per unit area or volume. It is expressed in watts per square meter (W m^−2^) (projected area of total anode surface) or in watts per cubic meter (W m^−3^) (anode chamber volume) [52];
- Coulombic efficiency (CE) is a parameter that represents the fraction of electrons obtained from oxidizable substrates that are recovered at the anode, which indicates the efficiency of converting a substrate to electrical energy [53]. In a BES, the coulombic efficiency is usually calculated as follows:

(1) $$CE=\frac{M\int_{0}^{t}Idt}{FbV_{An}\Delta COD}$$

where *M* = 32 is the molecular weight of oxygen, *I* is the electric current (calculated from the voltage generated by the BES), *F* = 96,485.33 C mol^−1^ is Faraday’s constant, *b* = 4 is the number of electrons exchanged per mole of oxygen, *V_An_* is the volume of the substrate in the anode chamber, and Δ*COD* is the difference in chemical oxygen demand (*COD*) over time [54].

## 3. Current Innovations in BES Technology

### 3.1. New Materials in Electrodes

The efficiency of BESs is contingent upon the composition of the electrode materials and the interaction between the electroactive bacteria and the surface of these components [55]. The following section describes some of the most innovative materials used to fabricate electrodes for BES applications:

- Carbon-based materials refer to a wide range of mainly carbon-based composite materials used in the synthesis and fabrication of electrodes. Of these, nanostructured carbonaceous matrices (e.g., carbon nanotubes, graphene, and mesoporous carbon) are of particular interest due to their high surface-to-volume ratio, excellent electrical conductivity, high biocompatibility, and low production costs [56]. Table 1 presents examples of these types of materials applied in BESs. According to these studies [57,58,59,60,61,62,63,64,65], using carbon-based materials has been demonstrated to enhance the fabricated electrodes’ electron transfer, surface area, and porosity. This factor is imperative for the augmentation of the performance of BES. Despite this, the authors note that the synthesis method influences the material properties, so the fabrication process selection must consider the specific application and system requirements;

- Metallic nanoparticles are particles measuring between 1 and 100 nm in size. They are utilized to fabricate electrodes in BESs to enhance extracellular electron transfer and electricity generation. These particles are typically applied in the form of coatings (on carbon-based materials or metals such as stainless steel), incorporated into nanocomposites, and utilized in the fabrication of 3D nanostructures (such as nanowires and nanospheres) [66,67]. Incorporating metal nanoparticles in electrodes augments their surface area and electrical conductivity while concurrently endowing these components with catalytic properties that can enhance oxidation–reduction reactions, thereby increasing the efficiency of BESs [68]. As shown in Table 2, Choi et al. [69], Sallam et al. [70], and Khandelwal et al. [71] utilized nickel (Ni), silver (Ag), and copper (Cu) nanoparticles, respectively, to engineer cathodes with electrocatalytic properties, thereby enhancing the oxygen reduction efficiency and consequently the overall system performance. On the other hand, Matsena et al. [72] and Tahir et al. [73] employed palladium (Pd) and nickel (Ni) nanoparticles, respectively, to increase the surface area and the electrical conductivity of carbon-based anodes. This significantly increased the performance of the studied BESs;

- Conductive polymers are organic compounds that can conduct electricity and thus improve electron transfer efficiency between electroactive microorganisms and electrodes in BESs. These materials have been shown to augment the surface area of the electrode, thereby providing additional sites for the adhesion of EAMs. The most commonly used conductive polymers include polyaniline (PANI), poly (3,4-ethylenedioxythiophene) (PEDOT), and polypyrrole (PPy), among others [74]. Table 3 presents examples of these types of materials applied in BESs. The extant literature on the subject agrees that the combination of conductive polymers with carbon-based materials (graphene oxide [75,76], carbon cloth [77], graphite felt [78], and carbon brush [79]) produces a synergistic effect that improves the conductivity, biocompatibility, and, in general, the energy efficiency of bioelectrochemical systems. Also, the authors mention that the surface modification of the electrodes increases their surface area and their roughness, which promotes microbial adhesion and electron transfer.

### 3.2. Development of New Microbial Strains

Several strategies have been described for developing new microbial strains for their use in bioelectrochemical systems (BESs):

- Genetic engineering and synthetic biology allow the modification of microorganisms to provide them with non-native functions or improve existing ones. These techniques can optimize extracellular electron transfer, which is crucial to increase the efficiency of BESs. Furthermore, they enable the incorporation of new enzymatic pathways, promoting the synthesis, detection, or oxidation of value-added products [80]. In this regard, Rabiço et al. [81] developed a novel exoelectrogenic strain of *Pseudomonas*, designated BJa5, and assessed its performance in a dual-chamber microbial fuel cell. This strain demonstrated a maximum power density of 39 mW m^−2^, a phenomenon attributed to its capacity to produce novel redox mediators. Conversely, Askitosari et al. [82] generated a novel *Pseudomonas putida* strain that expresses phenazine genes (heterocyclic compounds that function as natural redox mediators) from three distinct genetic sources of *Pseudomonas aeruginosa* for utilization in BESs. The authors observed that the developed strain generated four times more phenazines than the base strain, allowing this microbial species to be used in BESs. Furthermore, Fang et al. [83] engineered a mutant strain of *Geobacter sulfurreducens* that demonstrated an augmented capacity for producing outer membrane vesicles (OMVs), crucial for electron transfer in EAMs. This mutant strain also exhibited an enhanced ability to generate electric current in BESs;
- Selection and discovery of new electroactive strains involve identifying and characterizing microorganisms with a high capacity to transfer electrons out of the cell and their subsequent application to enhance the performance of BESs. This can be achieved by electrochemical enrichment and selection (controlling the anode potential and modulating biofilm formation) and the search for new electroactive microorganisms (such as some Gram-positive bacteria, extremophilic microorganisms, and cable bacteria) [84]. For instance, Hubenova et al. [85] identified a novel Gram-positive bacterial strain (genus *Paenibacillus*) capable of forming electrochemically active biofilms. The authors implemented this microbial strain in a BES, yielding positive outcomes (electric current of 200 mA m^−2^). Additionally, Narcizo et al. [86] isolated a novel strain of *Pseudomonas aeruginosa* that produces pioverdin (an iron-transporting siderophore) and evaluated its electrogenic capacity in a microbial fuel cell (MFC) fed with glycerol as a substrate. This strain was found to be a promising biocatalyst for bioelectricity production, generating an electric current similar (82.4 mA m^−2^) to other *P. aeruginosa* strains reported in the literature. Conversely, Ai et al. [87] effectively recovered both cupric ion and cadmium ion from acidic mining waters using a bioelectrochemical system inoculated with a novel exoelectrogenic strain of the genus *Pseudomonas* (designated as E8). The findings indicate that this strain possesses considerable potential for the treatment of acid mining waters, the recovery of heavy metals, and the generation of electrical energy, with a maximum power density of 70.40 mW m^−2^.

### 3.3. Technological Innovations for Large-Scale Applications

Implementing BESs on a large scale is contingent upon technological innovations that address the prevailing technical and economic challenges. Among the most notable advancements are the following: (1) A decline in the cost of construction materials, particularly exchange membranes and electrodes [88], (2) An enhancement in the energy efficiency of the systems through the intensification of bioelectrochemical processes and the optimization of operating conditions [89], (3) An integration with other technologies, such as direct osmosis, reverse electrodialysis, and pressurized filtration [90], (4) The development of mathematical models for automated BES design [91], and (5) A scale-up to pilot and industrial plant levels [92], among others.

## 4. Challenges in Scaling up BESs

### 4.1. Energy Efficiency

The energy efficiency of a BES depends on its capacity to convert the chemical energy present in the substrate to electricity. For the large-scale application of these systems to be successful, BESs must be able to generate high power densities at low operating costs [93]. However, low energy efficiency is a persistent problem in these systems. This phenomenon is attributable to various factors, including the system’s internal resistance, voltage losses (due to methanogenic processes in the anode chamber, electrode polarization and corrosion, and adverse operating conditions), and mass transfer limitations (substrate availability, biofilm thickness, product accumulation at the anode, and oxygen diffusion at the cathode) [94,95].

One of the biggest challenges in BES scale-up is decreased energy efficiency with augmenting reactor size [96]. This is partly due to the increase in ohmic resistance as reactor components increase in size. Large-scale systems are also prone to voltage losses due to increased electrode spacing and exchange membrane surface area, which facilitates oxygen diffusion into the anode chamber [97,98].

### 4.2. Cost Considerations

When considering BES scale-up, costs are a decisive factor. Multiple aspects contribute to the total cost, from construction materials to system maintenance. Electrodes (made from materials such as gold or platinum) and exchange membranes represent a substantial expense in the manufacturing of these systems [99]. Also, these components have a limited lifetime and need to be replaced periodically [100].

Conversely, the operational expenditures may fluctuate due to various factors, including energy consumption (external power supply is often necessary for the aeration of the cathodic chamber and potential control in the BES), the pretreatment of highly complex substrates (which may be required to remove substances that inhibit microbial activity or damage reactor components), and labor (large-scale BES operation necessitates specialized personnel for monitoring, maintenance, and troubleshooting) [101,102,103].

### 4.3. Biocatalysts and Microbial Community Stability

The large-scale implementation of BESs faces challenges related to biocatalysts and the stability of microbial communities. Two key aspects that impact their performance are the efficiency and stability of the electroactive biofilm (maintaining a stable biofilm at a large scale can be difficult due to factors such as hydrodynamic conditions, temperature and pH variations, and nutrient availability) and microbial competition (in mixed culture systems, competition between electroactive and non-electroactive microorganisms can lead to a decrease in electron transfer efficiency and energy production) [104,105]. Consequently, developing robust and stress-tolerant EAMs is imperative to ensure the sustainability of BESs as a technology.

## 5. Case Studies in Scaling up BESs

Notwithstanding the present limitations, several authors have effectively scaled up bioelectrochemical systems. For instance, Valladares-Linares et al. [106] assessed a system comprising 18 stacked microbial fuel cells (with a total volume of 700 L) for domestic wastewater treatment. The outcomes of this study demonstrated that the pilot plant-scale BES can operate continuously without external energy and can significantly improve the quality of the treated effluent. Furthermore, Goto and Yoshida [107] investigated organic matter removal in slaughterhouse wastewater using single-compartment microbial fuel cells with varying volumetric capacities (1.5 L, 12 L, and 100 L). The authors highlighted that the pilot plant scale MFC (100 L) produced a maximum power density and average organic matter removal of 2.1 Wh m^−3^ and 52%, respectively, thus suggesting that this type of technology can be used for the remediation of meat industry effluents. Conversely, Guerrero-Sodric et al. [108] operated a pilot plant-scale double chamber microbial electrolysis cell (135 L) for six months, during which they modified the operational conditions (applied potential, hydraulic retention time, temperature, and substrate) to ascertain the limiting factors in hydrogen production. The authors concluded that at an applied potential of 0.9 V, temperatures above 30 °C, HRT of 1 d, and using synthetic wastewater as substrate, the system generated a maximum power density of 1.23 A m^−2^ and produced 0.1 m^3^ m^−3^ d^−1^ of hydrogen. This demonstrated the capacity of the BES to treat polluted effluents and sustainably produce hydrogen. In a related study, Huang et al. [109] examined the recovery of heavy metals (Cu^2+^, Ni^2+^, Zn^2+^, and Cr^6+^) and the removal of recalcitrant organic compounds in a pilot plant-scale cylindrical microbial electrolysis cell (40 L) fed with terminal etching wastewater at varying hydraulic retention times. The study demonstrated the complete recovery of the evaluated heavy metals and efficient treatment of real etching terminal wastewater. This offers a viable method for the simultaneous recovery of value-added products and treating highly contaminated effluents. In addition, Das et al. [110] designed and operated a pilot plant-scale (12.6 L) bioelectrochemical system for the production of acetic acid from biogas (as a carbon source). The system demonstrated the capacity to produce up to 70.55 g m^−2^ of acetic acid daily, exhibiting a coulombic efficiency of 77.8%. This outcome substantiates the viability of the proposed technology for carbon capture and its conversion into valuable compounds. Table 4 provides a synopsis of the most current case studies about the scale-up of bioelectrochemical systems. In these papers [111,112,113,114,115,116,117,118,119,120,121,122,123,124,125,126,127,128,129,130,131], the authors agree that scaling BESs remains a significant challenge. They note that the transition is not straightforward and that a decrease in performance is often observed with increasing reactor size. The authors also emphasize that optimizing several factors is critical to improving the performance of BESs at larger scales. These factors include electrode design and spacing [112,114,128], the selection of exoelectrogenic microorganisms compatible with the substrate and reactor operating conditions [123], selected hydraulic retention time [117], applied voltage (in the case of MECs) [129], and temperature [124], among others. Finally, Guerrero-Sodric et al. [111], Chen et al. [115], and Rossi et al. [125] highlight the need for comprehensive technical and economic analyses as well as life cycle assessments to determine the industrial feasibility and environmental impact of large-scale BESs.

## 6. Future Directions and Emerging Trends

### 6.1. Circular Economy Principles in BESs

The BES paradigm fosters a transition from a linear economic model (extract, manufacture, use, and discard) to a circular system that emphasizes the reuse of resources, the reduction of waste, and the closure of material cycles. By these principles, and as previously discussed, these systems facilitate the recovery of resources (e.g., nutrients, metal ions, and water), decentralized energy generation (mainly in rural or off-grid communities), the generation of value-added products (e.g., biofuels, biochemicals, and biomaterials), and the integration of processes (e.g., coupling this type of technology with anaerobic digestion systems, membrane bioreactors, etc.) [132,133,134].

### 6.2. Hybrid BESs

Bioelectrochemical hybrid systems represent a combination of conventional BESs with other technologies to increase efficiency and overcome the limitations of stand-alone systems [135,136]. These systems include plant MFCs (phyto-assisted bioremediation combined with MFC technology), microbial solar cells (integrate photoautotrophic and electrochemically active microorganisms to generate green electricity), microbial-enzymatic fuel cells (use enzymes to increase the rate of electron transfer by improving the current density produced), microbial reverse-electrodialysis cells (generate electricity from salinity gradients), and bio-electrochemical constructed wetland systems (favor the treatment of contaminated effluents and the generation of electrical energy through the interaction of EAMs, plants, and the endemic microbiota of the wetland), among others [137,138]. While these systems show promise, their large-scale implementation is limited by economic feasibility, long-term stability, and the complexity of fabrication and operation [139].

## 7. Conclusions

The constant population growth has dramatically increased the energy demand globally. Bioelectrochemical systems (BESs) offer a sustainable source for generating clean energy, treating contaminated water and soil, and producing high-value-added products. To date, significant progress has been made in developing new materials for the manufacture of electrodes, the discovery and development of new electroactive microbial strains, the reduction of construction material costs, and, in general, various factors that facilitate the scaling up of these types of systems. However, significant challenges persist (low energy efficiency, high fabrication and construction costs, and poor microbial community stability) that limit the effective transition of BESs from the laboratory to large-scale application. Advances in the engineering of these systems, new optimization strategies, the development and implementation of hybrid bioelectrochemical systems, and a circular economy approach could be key to greater adoption and success of these technologies at industrial scales.

## Figures and Tables

**Table 1:** Carbon-based materials recently used in BESs.

Anode	Cathode	CEM	Microorganisms	Substrate	Performance	Reference
Carboxylated multiwalled carbon nanotubes/carbon nanofibers composite	Carbon cloth	Manufacturer not specified	Preacclimated bacteria from a sediment of a freshwater wetland	Artificial wastewater	362 ± 20 mW m^2^	[57]
Carbon cloth modified with carbon nanotubes (CNTs)	Carbon cloth with a platinum covering	Nafion 117	Provided by the substrate	Municipal wastewater	393.8 mW m^−2^	[58]
Carbon felt	CNTs hybridized molybdenum disulfide nanocomposites	Nafion 117	Bacteria originated from a stable operating MFC	Sodium acetate	53.0 mW m^−2^	[59]
3D graphene structures	Stainless steel mesh	Nafion 117	Anaerobic pre-treated sludge	Synthetic wastewater	0.49 mW m^−2^	[60]
Nitrogen-doped graphene oxide	Carbon cloth	Nafion 117	Anaerobic activated sludge	Acetate-based solution	708 mW m^−2^	[61]
Reduced graphene oxide decorated with Cu_2_O nanoparticles	Carbon cloth	Membraneless	Activated sludge	Acetate-based solution	Presented as voltage (0.223 V)	[62]
Carbon felt decorated with graphitic mesoporous carbon	Carbon paper loaded with Pt	CMI 7001	Anaerobic digester sludge	Acetate-based solution	70.3 mW m^−2^	[63]
Carbon paper	Carbon cloth covered with a nitrogen- and phosphorous-doped, ordered mesoporous carbon	PTFE diffusion layer	Anaerobic digester sludge	Acetate-based solution	245.8 mW m^−2^	[64]
Carbon felt	Nitrogenous mesoporous carbon coated with Co and Cu nanoparticles	Membraneless	Domestic wastewater	Acetate-based solution and domestic wastewater	2033 mW m^−2^	[65]

**Table 2:** Metallic nanoparticles recently used in BESs.

Anode	Cathode	CEM	Microorganisms	Substrate	Performance	Reference
Carbon felt	Carbon felt loaded with Ni nanoparticles	CMI-7000	Anaerobic digester sludge	Anaerobic nutrient solution with acetic acid as carbon source	1630.7 mW m^−2^	[69]
Graphite based (characteristics not specified)	Ag nanoparticle-activated carbon composite	CMI-7000	Provided by the substrate	Seawater	2500 mW m^−2^	[70]
Graphite felt	Cu nanoparticle-activated graphite composite	Ultra-filtration membrane	Cow manure	Fruit pulp	6000 mW m^−3^	[71]
Biogenic Pd nanoparticles loaded on a carbon rod	Carbon rod	Nafion 117	Wastewater treatment plant sludge	Basal mineral medium with glucose or sodium formate as carbon source	4.01 mW m^−2^	[72]
Nickel ferrite nanoparticles/MXene-coated carbon felt	Carbon cloth	Nafion 117	Wastewater treatment plant sludge	Growth media (characteristics not specified)	1385 mW m^−2^	[73]

**Table 3:** Conductive polymers recently used in BESs.

Anode	Cathode	CEM	Microorganisms	Substrate	Performance	Reference
Graphene–polyaniline composite	Graphite rod	Not specified	Treated wastewater	Treated wastewater and sweet potato	0.0016 mW m^−2^	[75]
Stainless steel/PEDOT/graphene oxide	Graphite rod	CMI-7000	*Methanococcus deltae*	Glucose	1014.42 mW cm^−2^	[76]
Carbon cloth	NiCo_2_O_4_/PANI/carbon cloth	Nafion 117	*Pseudomonas aeruginosa*	Synthetic wastewater with an azo dye	12.19 mW m^−2^	[77]
Polydopamine/polypyrrole -graphite felt	Graphite rod	Nafion 117	Anaerobic digester sludge	Acetate-based solution	929 mW m^−2^	[78]
Polypyrrole-carboxymethyl cellulose-carbon nanotube/carbon brush	Graphite rod	Proton exchange membrane (type not specified)	Electricity-producing microorganisms (origin was not specified)	Acetate-based solution	2970 mW m^−2^	[79]

**Table 4:** Overview of recent case studies in scaling up BESs.

Type of BES	Total Volume	Anode	Cathode	CEM	Microorganisms	Substrate	Performance	Reference
MEC	150 L	Carbon felt	Ni-foam	RALEX^®^	Anaerobic sludge	Diluted industrial wastewater	Presented as current density (2 A m^−2^)	[111]
MEC	88 L	Carbon felt	Stainless-steel wire wool	Rhinhode	Provided by the substrate	Domestic wastewater	Presented as current density (0.3 A m^−2^)	[112]
MEC	10 L	Carbon cloth	Carbon cloth coated with MoP	Membraneless	Mixed bacteria culture from another MEC	Acetate based solution	Presented as current density (970 A m^−3^)	[113]
MEC	168 L	Reticulated vitreous carbon	Reticulated vitreous carbon-Pt	Nafion 117	Provided by the substrate	Municipal wastewater	Not reported	[114]
MEC	130 L	Stainless-steel mesh wrapped with graphite fibers	Stainless-steel wire	Anion exchange membrane (brand not specified)	Provided by the substrate	Pre-treated urban wastewater	Presented as current density (270 mA m^−2^)	[115]
MEC	15 L	Carbon tissue strips with a stainless-steel frame	Granular carbon	Fumatech	From a carbon-tissue-bioanode running on an H-type reactor	Biowaste hydrolysate	Presented as current density (10.5 A m^−2^)	[116]
MEC	72 L	Carbon felt	Stainless-steel wire	Rhinhode	Return sludge liquor and effluent of an operating MFC	Return sludge liquor	Presented as current density (1.12 A m^−2^)	[117]
MEC	16 L	Graphite felt	Stainless-steel mesh	CMI-7000	Digestate from a wastewater treatment plant	Pig slurry	Presented as current density (1.75 A m^−2^)	[118]
MFC	1200 L	Carbon fabric	Carbon fabric	Membraneless	Anaerobic mixed culture	Raw municipal wastewater	8.8 mW m^−2^	[119]
MFC	65 L	Graphite gravel	Graphite gravel	Membraneless	Digested biogas slurry	Synthetic wastewater	11.67 mW m^−3^	[120]
MFC	316 L	Graphite plate	Activated carbon	Membraneless	Provided by the substrate	Pond water	Presented as voltage (450 mV)	[121]
MFC	28 L	Carbon felt	Carbon felt	Membraneless	Anaerobic sludge	Synthetic wastewater	129 mW m^−2^	[122]
MFC	125 L	Carbon felt	Carbon felt coated with CuSn	Clayware ceramic	Anaerobic sludge	Septic tank slurry	83 mW m^−2^	[123]
MFC	85 L	Graphite fiber	Activated carbon	Membraneless	Provided by the substrate	Domestic wastewater	0.101 W m^−2^	[124]
MFC	1400 L	Carbon fiber brush	Activated carbon	Membraneless	Provided by the substrate	Domestic wastewater	0.043 W m^−2^	[125]
MFC	25 L	Carbon felt	Carbon felt coated with CuZn nanoparticles	Membraneless	Anaerobic sludge	Sewage sludge slurry	7.5 W m^−3^	[126]
MES ^1^	5.86 L	Steel mesh covered with carbon powder	Carbon cloth	Nafion 117	Activated sludge	Synthetic wastewater	Presented as current density (0.002 mA cm^−2^)	[127]
MES ^1^	1500 L	Graphite fiber brush	Graphite fiber brush	Membraneless	Primary sedimentation tank effluent from a wastewater treatment plant	Domestic and industrial wastewater	406 mW m^−3^	[128]
EMG-BES ^2^	32 L	Activated carbon	Activated carbon	Membrane-less	Anaerobic sludge	Municipal wastewater	Presented as current density (0.5 A m^−2^)	[129]
EMG-BES ^2^	50 L	Carbon laying	Carbon fabric	FKSPET-130	*Methanococcus maripaludis* S2	Sterile-filtrated MES medium	Presented as current density (85 mA m^−2^)	[130]
GCMB-BES ^3^	7.7 L	Activated carbon granules	Titanium mesh coated with Pt/Ir	RALEX^®^	Mixed electroactive community from an operation MEC	Acetate-based solution	Presented as current density (23 A m^−2^)	[131]

^1^ Microbial electrosynthesis cell. ^2^ Electromethanogenesis bioelectrochemical system. ^3^ Granular capacitive moving-bed bioelectrochemical system.

## Data Availability

No new data were created or analyzed in this study.
